# A Case of Erythrocytosis in a Patient Treated with an Aromatase Inhibitor for Breast Cancer

**DOI:** 10.1155/2013/615189

**Published:** 2013-11-07

**Authors:** Abhinav Iyengar, Dawn Sheppard

**Affiliations:** Hematology Division, Department of Medicine, The Ottawa Hospital, 501 Smyth Road, Ottawa, ON, Canada K1H 8L6

## Abstract

A previously healthy 79-year-old female was referred to hematology for further evaluation of erythrocytosis. Two years earlier she had been diagnosed with ER/PR-positive ductal carcinoma of the breast and was receiving hormonal therapy with exemestane. No secondary cause of erythrocytosis was identified. Serum erythropoietin (EPO) level was normal, and molecular testing for the JAK2 V617F and exon 12 mutations was negative. A bone marrow biopsy showed a mild increase in erythropoiesis, and no spontaneous erythroid colonies were demonstrated. Erythrocytosis is common reason for referral to a hematologist. The myeloproliferative disorder, polycythemia vera, and the rare congenital polycythemias represent primary erythrocytosis. Common secondary causes include smoking, obstructive sleep apnea, and other pulmonary diseases. Erythrocytosis is well described with certain classes of drugs, including androgens. We hypothesize that exemestane contributed to the development of erythrocytosis in our patient. To our knowledge, erythrocytosis has not been previously described in association with aromatase inhibitors. These drugs prevent the conversion of androstenedione and testosterone to estrogen; thus the physiologic mechanisms may be similar to those responsible for erythrocytosis seen with exogenous androgens. These mechanisms are not well understood, but may include altered iron metabolism by a reduction in hepcidin levels.

## 1. Case Presentation

A 79-year-old female was referred to a hematologist for evaluation of erythrocytosis. In late 2009, she was diagnosed with HER2-positive T1_C_N_0 _M_0_ infiltrating ductal carcinoma of the left breast, which was treated with wide local excision, four cycles of chemotherapy with docetaxel and cyclophosphamide, radiation, and trastuzumab. Her tumour was ER/PR-positive, and so letrozole was started as adjuvant hormone blocking therapy. Letrozole was discontinued after a few months due to nausea. She subsequently started exemestane 25 mg daily in September 2010. Prior to commencing exemestane her hemoglobin and hematocrit were normal at 154 g/L and 44.1%, respectively. Her MCV was 88.7 fL, and her other blood counts were normal.

Her oncologist thereafter noted a gradual increase in her hematocrit, and she was referred for hematologic evaluation. She was seen in consultation in November 2011. At that time, she denied headache, visual changes, erythromelalgia, or other vasomotor symptoms. There were no neurological symptoms. She acknowledged mild pruritus associated with seasonal allergies. She denied fevers, night sweats, or weight loss. There was no history of peripheral edema, chest pain, dyspnea, or cough. She denied abdominal pain or changes in bowel habit. She had no urinary symptoms. 

Her past medical history included asthma, hypertension, hypothyroidism, and osteopenia. She denied previous thrombotic or hemorrhagic events. At the time of consultation, her medications included fluticasone, salbutamol, amlodipine, levothyroxine, risedronate, and exemestane. 

On examination, there was facial plethora, but she otherwise appeared well. There was no evidence of volume contraction. There was no hirsutism or other signs of virilization. Her blood pressure was 150/90, heart rate 88 beats per minute, and oxygen saturation 93%. She had no peripheral lymphadenopathy. Her cardiac examination revealed a normal JVP with normal heart sounds and no extra sounds, murmurs, or gallops. Peripheral pulses were normal in all four extremities. Her chest was clear on auscultation and there were no signs of clubbing or cyanosis. Her abdomen was soft with no palpable masses or hepatosplenomegaly. 

Her hemoglobin and hematocrit at that time were 187 g/L and 53.6%, respectively. Her white count was 5.9 × 10^9^/L, and her platelet count was 215 × 10^9^/L. Hepatic enzymes and LDH were normal. Creatinine was 71. She had never received a red cell transfusion and did not have evidence of iron overload. Iron studies showed a ferritin of 111 *μ*g/L (11–307 *μ*g/L), serum iron of 25 *μ*mol/L (9–30 *μ*mol/L), and total iron binding capacity of 73 *μ*mol/L (45–81 *μ*mol/L). Her chest X-ray showed mild hyperinflation, and pulmonary function testing revealed mild obstruction. Abdominal ultrasound was unremarkable. Serum erythropoietin (EPO) level was normal at 3.5 IU/L (2.6–18.5 IU/L). This was confirmed on a second occasion. Molecular testing for the JAK2 V617F and exon 12 mutations, as well as for the BCR-ABL1 translocation, was negative. A bone marrow biopsy showed a mild increase in erythropoiesis but was otherwise normal with no increase in granulopoiesis, megakaryopoiesis, or increased blasts. Erythroid cultures were performed to look for spontaneous erythroid colony formation in the absence of erythropoietin, but none were demonstrated. 

Her hematocrit peaked at 54.7% in March 2012, 18 months after starting exemestane, and she underwent five phlebotomies between March and May 2012 with a resultant decrease to 42.9%. However, it was noted that the development of her erythrocytosis correlated with the initiation of the aromatase inhibitor, exemestane. Exemestane was discontinued in May 2012. She has had no phlebotomies since that time, and there has been no recurrence of erythrocytosis. 

## 2. Discussion

### 2.1. Regulation of Erythropoiesis

Under physiologic conditions, EPO production is upregulated in the setting of decreased oxygen delivery to tissues. Hypoxia results in the production of hypoxia-inducible factor (HIF)-1, the major transcription factor responsible for activation of the EPO gene [1]. EPO is produced primarily in the kidney and interacts with the EPO receptor on erythroid progenitor cells. When EPO binds, the EPO receptor interacts with JAK2, resulting in phosphorylation of itself and STAT5 [2]. The JAK2/STAT5 signaling pathway plays an important role in erythroid development. 

### 2.2. Differential Diagnosis of Erythrocytosis

Erythrocytosis is a common referral to the hematologist. The first step in evaluation is to determine whether the erythrocytosis is relative or absolute (Box 1). Relative erythrocytosis occurs when there is plasma volume contraction. Absolute erythrocytosis results from increased red cell mass. Absolute erythrocytosis may be primary, most commonly polycythemia vera (PV), or secondary. Secondary erythrocytosis may result from hypoxic conditions, including cyanotic heart disease, pulmonary disease, carbon monoxide (e.g., smokers), obstructive sleep apnea, and high altitude. These conditions are associated with a serum EPO level that is appropriately increased.

Secondary erythrocytosis may also result from conditions associated with an inappropriately elevated serum EPO. These include EPO-secreting tumours, such as renal cell carcinoma, hepatocellular carcinoma, and uterine leiomyomata, and following renal transplantation. Several medications, including recombinant erythropoietin and androgens, may result in erythrocytosis. Rare congenital causes include hemoglobin mutations resulting in increased oxygen affinity, EPO receptor mutations, and mutations affecting regulation of EPO production by HIF, for example, Chuvash polycythemia.

Determining the cause of erythrocytosis requires consideration of a patient's risk factors for secondary erythrocytosis. A careful history and physical and repeated documentation of abnormal blood values are quite helpful. Measurement of oxygen saturation can determine if hypoxia is present. In addition, a smoking history or history of sleep disordered breathing may help in determining if chronic obstructive lung disease or obstructive sleep apnea is present. Chest radiography, pulmonary function testing, or polysomnography may be warranted for confirmation. A history of congenital heart disease or renal disease is also important. Abdominal ultrasound may be helpful to rule out an EPO-producing tumour. Once these factors have been eliminated, investigations looking at primary causes should be considered.

### 2.3. Polycythemia Vera

PV is the most common of the classical myeloproliferative neoplasms. It is a clonal disorder characterized by splenomegaly, a predisposition to thrombohemorrhagic events, and an increased risk of progression to acute leukemia. The diagnosis of PV is aided by the testing for the JAK2 V617F mutation, which is typically positive in over 90% of PV cases [3]. The discovery of this mutation has been a major advance in the diagnosis of myeloproliferative disorders. The use of serum EPO levels can also be useful in elucidating the cause of erythrocytosis. Endogenous EPO levels should be suppressed or normal in PV. Elevated EPO levels in the setting of erythrocytosis signify either appropriate compensation, an exogenous source of EPO, or malignant production of EPO. 

The diagnosis of PV can be difficult, especially in the absence of the JAK2 V617F mutation or any identifiable secondary causes. Testing for mutations in JAK2 exon 12 can also be performed in cases where the more common JAK2 V617F mutation is absent. Exon 12 mutations have been reported to be present in 80% of those with PRV who are JAK2 V617F negative [3]. Cultures of erythroid progenitors in PV patients demonstrate spontaneous erythroid colony formation in the absence of EPO. This autonomous proliferation is the hallmark of PV. 

### 2.4. Possible Association between Erythrocytosis and Aromatase Inhibition

Our case involves absolute erythrocytosis in the absence of a definitive primary disorder. The absence of JAK2 V617F and exon 12 mutations in addition to the lack of spontaneous erythroid colony formation pointed to a secondary cause of the erythrocytosis. The timing from the initiation of exemestane to the first appearance of erythrocytosis was highly suspicious. 

A literature search was performed using Medline, PubMed, and Google Scholar with the terms polycythemia, erythrocytosis, aromatase inhibitors, and the names of commonly used aromatase inhibitors, both drug and brand names. We found one report of two patients treated with letrozole who developed erythrocytosis [4].

The use of antiestrogen therapy in ER-positive breast cancer is a mainstay of treatment. Evidence for the use of aromatase inhibitors in postmenopausal women in the metastatic, neoadjuvant, and adjuvant setting is increasing. Exemestane is a steroidal aromatase inhibitor that prevents the conversion of androstenedione and testosterone to estrogen in peripheral tissues. The prescribed dose in the adjuvant setting is 25 mg daily. Dosing trials of exemestane showed increases in testosterone at doses in excess of 200 mg daily. 

Erythrocytosis is a common side effect in males on testosterone replacement. The exact mechanism by which testosterone causes erythrocytosis is not known. Most recently, a study looking at the impact of testosterone on hepcidin, a key component in iron metabolism, showed that exogenous testosterone decreased hepcidin levels [5]. Decreases in hepcidin levels were shown to correlate with increases in hemoglobin and hematocrit. 

We hypothesize that the mechanism of exemestane causing erythrocytosis may be related to physiologic increases in androgen levels as consequence of aromatase inhibition. The increased androgens, much like exogenous testosterone, lead to erythrocytosis, which may or may not be related to the mechanism of decreased hepcidin levels. The temporary use of phlebotomies and cessation of exemestane in our case led to the resolution of the erythrocytosis. It is impossible to prove causality in this case; however, there was a clear temporal relationship, and the erythrocytosis worsened with continuation and resolved with discontinuation of the drug. There was no evidence of polycythemia vera, and a thorough workup failed to reveal an alternative secondary cause. Finally, the mechanism of erythrocytosis is biologically plausible. Although a rechallenge with exemestane would have strengthened the association, this was not acceptable to the patient and was not performed. A follow-up period of 12 months in the absence of phlebotomies has not shown any recurrence of erythrocytosis. Given the widespread use of this drug, this may represent an idiosyncratic reaction not previously seen. 

## Figures and Tables

**Box 1:**
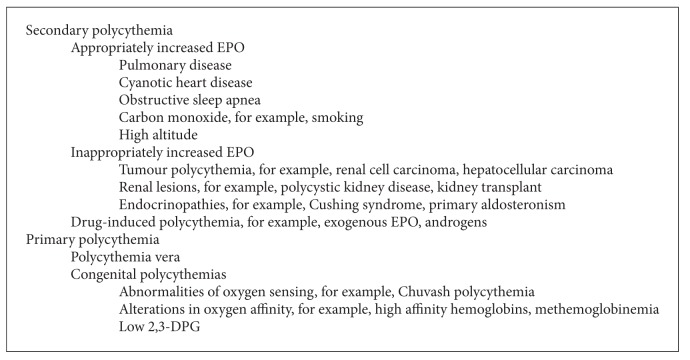
Differential diagnosis of erythrocytosis. EPO indicates erythropoietin; 2,3-DPG, 2,3-disphosphoglycerate.
